# P-2151. Incidence of Breakthrough HSV in Adult Allogeneic Hematopoietic Cell Transplant Recipients on Standardized Antiviral Prophylaxis

**DOI:** 10.1093/ofid/ofaf695.2314

**Published:** 2026-01-11

**Authors:** Ria Mohan, Christine Johnston, Amanda Phipps, Chris Davis, Agnes Ho, Michael J Boeckh, Emily Ford, Denise McCulloch, Ted Gooley, Frank P Tverdek, Steven A Pergam, Molly D Fischer

**Affiliations:** Virginia Commonwealth Univeristy, Richmond, VA; University of Washington, Seattle, WA; University of Washington, Seattle, WA; Fred Hutchinson Cancer Research Center, Seattle, Washington; Fred Hutchinson Cancer Center, Seattle, Washington; Fred Hutchinson Cancer Center, Seattle, Washington; Fred Hutchinson Cancer Center, Seattle, Washington; Fred Hutchinson Cancer Center, Seattle, Washington; Fred Hutchinson Cancer Center, Seattle, Washington; Fred Hutchinson Cancer Center / University of Washington, bellevue, WA; Fred Hutchinson Cancer Center; University of Washington, Seattle, WA; University of Washington, Seattle, WA

## Abstract

**Background:**

Reactivations of herpes simplex viruses (HSV) can occur in the early post-allogeneic hematopoietic cell transplant (aHCT) period despite universal antiviral prophylaxis. Few studies have assessed HSV recurrence in the era of standardized antiviral prophylaxis, in which val/acyclovir is recommended for up to 1 year post aHCT. We evaluated the incidence and management of HSV during the first 100 days post-HCT over two decades.

**Figure 1:**
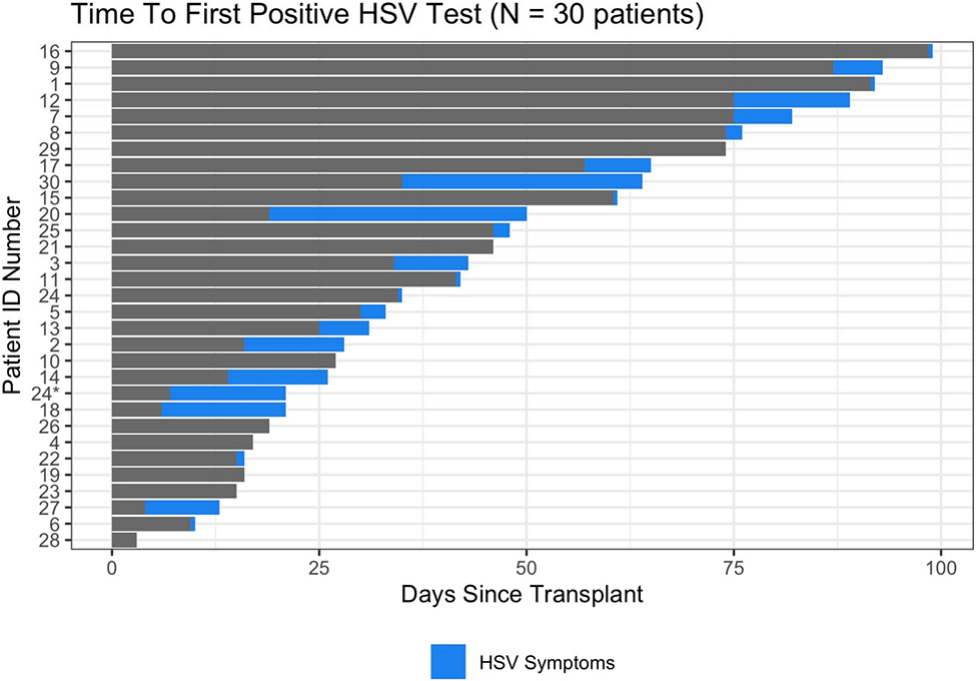
Time from transplant to first positive HSV test and development of symptoms

**Methods:**

All first aHCT recipients at Fred Hutchinson Cancer Center between 2002-2022 were reviewed to determine the incidence of HSV within the first 100 days on prophylaxis (acyclovir 800 mg or valacyclovir 500 mg twice daily). HSV cases were identified via viral culture, polymerase chain reaction, and/or direct fluorescent antibody testing, and clinical records were reviewed for symptoms, clinical outcomes, use of prophylaxis, and treatment regimens. Resistance was categorized as clinical (presumptive) or virologically-confirmed by the University of Washington Virology laboratory.

**Results:**

We reviewed data from 4,358 aHCT recipients aged ≥18 years, among whom 3,749 (86%) were HSV seropositive and 30 developed HSV recurrence (cumulative incidence = 0.8%). Most (n = 22) reactivations were HSV-1 and 8 were HSV-2; 2 were unspecified. The median time from transplant to first positive test was 35 days (IQR: 20-65 days) (Figure 1). HSV was detected at multiple anatomic sites; oral recurrences were most common. In total, 14/30 (46.7%) patients developed acyclovir-resistant HSV (8 virologically-confirmed, 6 clinical). Treatment duration was significantly longer for patients with resistant (median 38 days [IQR: 26-41]) compared to susceptible infections (20 days [IQR: 16-27]; p = 0.03). Few patients (n = 4) had events attributed to non-adherence/malabsorption.

**Conclusion:**

Clinical HSV disease is rare among aHCT patients on prophylaxis in the first 100 days. Development of acyclovir resistance is uncommon but represented almost half of breakthrough cases. Our findings highlight the importance and effectiveness of universal val/acyclovir prophylaxis in the early post-transplant period.

**Disclosures:**

Christine Johnston, MD, MPH, AiCuris: Advisor/Consultant|Assembly Biosciences: Advisor/Consultant|GlaxoSmithKline: Advisor/Consultant|GlaxoSmithKline: Grant/Research Support|Moderna: Grant/Research Support|Pfizer: Advisor/Consultant Michael J. Boeckh, MD PhD, Allovir: Advisor/Consultant|Ansun Biopharma: Grant/Research Support|AstraZeneka: Advisor/Consultant|AstraZeneka: Grant/Research Support|GSK: Grant/Research Support|Merck: Advisor/Consultant|Merck: Grant/Research Support|Moderna: Advisor/Consultant|Moderna: Grant/Research Support|Symbio: Advisor/Consultant|Vir Biotechnology: Grant/Research Support Denise McCulloch, MD, MPH, Pfizer: Grant/Research Support Steven A. Pergam, MD, MPH, F2G: Site PI for clinical trial|Global Life Technologies, Inc.: Grant/Research Support|Mundipharma: Site PI for clinical trial|Symbio: Site PI for clinical trial

